# The Prevalence of Mental Health Conditions in Adults and Children With Childhood-Onset Inflammatory Ocular Disease: Protocol for a Systematic Review and Meta-Analysis

**DOI:** 10.2196/88888

**Published:** 2026-05-13

**Authors:** Sonali Dave, Asha Vanzara, Ameenat Lola Solebo

**Affiliations:** 1 UCL GOS Institute of Child Health Population, Policy and Practice Programme University College London London, England United Kingdom; 2 Great Ormond Street Hospital London, England United Kingdom; 3 University of Cambridge Cambridge, England United Kingdom; 4 Moorfields Eye Hospital NHS Foundation Trust London, England United Kingdom; 5 NIHR Great Ormond Street Hospital Biomedical Research Centre London, England United Kingdom

**Keywords:** inflammation, autoimmune diseases, mental health, ocular disease, eye disease

## Abstract

**Background:**

Inflammatory ocular diseases (IODs) are frequently associated with multisystem autoimmune conditions and can lead to substantial visual morbidity, including visual impairment and blindness. Emerging evidence suggests that inflammation contributes to the development of depression and other mental health disorders. Individuals with childhood-onset IOD may be at increased risk of poor mental health outcomes and quality of life. However, the prevalence of mental health conditions in this population remains unclear.

**Objective:**

This study aims to review the evidence regarding the prevalence of mental health conditions among children and adults with childhood-onset IOD.

**Methods:**

This systematic review will follow the PRISMA (Preferred Reporting Items for Systematic Reviews and Meta-Analyses) 2020 guidelines. Eligible studies will report mental health disorder prevalence and outcomes in individuals with childhood-onset IOD, regardless of age at assessment of outcome. Studies evaluating interventions or focusing primarily on mental health effects secondary to visual impairment or blindness will be excluded. Searches will be conducted in PubMed, the Cochrane Central Register of Controlled Trials, Embase, Ovid, and PsycArticles databases. Gray literature will be identified through Google searches. Two researchers will independently screen titles, abstracts, and full texts; extract data; and assess risk of bias using the risk of bias in nonrandomized studies of exposure tool; disagreements will be resolved by a third reviewer. Data will be synthesized descriptively, with attention to study design, outcome measures, cooccurrence of multisystem disease, and methodological quality.

**Results:**

A preliminary scoping search has been completed to estimate the volume of relevant literature. Full searches began in November 2025. Data collection and extraction is currently in progress. Data extraction, analysis, and synthesis will follow, using a narrative approach to summarize mental health outcomes across studies. The final review is expected to be completed by August 2026.

**Conclusions:**

The findings from this review will help to establish the prevalence of mental health conditions among people with childhood-onset IOD. The results from this review will support recommendations for further research and policies to ensure the best health outcomes for children.

**International Registered Report Identifier (IRRID):**

DERR1-10.2196/88888

## Introduction

Inflammatory ocular disease (IOD) encompasses a range of eye disorders in which inflammation is the main underlying process. These include conditions such as uveitis, keratitis, scleritis, conjunctivitis, retinitis, optic neuritis, orbital inflammatory disease, dry eye disease, and keratoconjunctivitis [1] (see Table 1 for more specific disease characteristics). All of these conditions involve inflammation in different areas of the eye and in many cases are associated with autoimmune conditions that manifest systemically or in extraocular organs. Given the complexity of these ocular diseases, patients may need frequent eye assessments; may require long-term monitoring, management, and treatment; and must often see several specialists simultaneously to manage their conditions. This can be complex and burdensome, particularly for children, young people, and their families. This burden is compounded by the rarity of the disorder, and the constant risk of future vision loss.

**Table 1:** Summary of the characteristics of inflammatory ocular diseases.

IOD^a^	Summary of the characteristics
Uveitis	A heterogeneous group of diseases characterized by inflammation in the eye. They can be infectious or noninfectious (typically immune mediated) and are characterized by the location of inflammation (anterior uveitis, intermediate uveitis, posterior uveitis, etc).
Keratitis	Inflammation in the cornea that can be due to infection, autoimmune conditions, or trauma.
Scleritis	Severe inflammation affecting the sclera, either anterior or posterior, often associated with autoimmune diseases.
Conjunctivitis	Infection or allergy-induced inflammation in the conjunctiva.
Retinitis	Inflammation of the retina that is often caused by viral or bacterial infections or autoimmune conditions such as sarcoidosis or Behcet disease and often associated with immunosuppression.
Optic neuritis	Inflammation of the optic nerves, frequently associated with multiple sclerosis and can be caused by autoimmune diseases or infections.
Dry eye disease	Characterized by a loss of tear film that can lead to ocular surface inflammation.
Orbital inflammation	Inflammation of the tissues within the eye socket that can be infectious or noninfectious and can be caused by autoimmune diseases, tumors, or trauma.
Keratoconjunctivitis	Inflammation of the cornea and conjunctiva that can be caused by viral or bacterial infections, allergies, and is commonly associated with autoimmune conditions.

^a^IOD: inflammatory ocular disease.

The positive association between the pathology of inflammation and major depressive disorder and anxiety in children, adolescents, and adults has been studied previously [2-6]; however, none of these studies specifically include IODs. Separately, there is a significant association between poor quality of life (QoL) and mental health conditions in blind or visually impaired children. They also have greater odds of experiencing depression and anxiety compared to individuals without visual impairment [7]. However, the underlying drivers of distress and psychological burden in people with visual impairment often include loss of function and independence, social isolation, and perceived and actual barriers to work or education [8-10]. This is different to children with IODs where the psychological burden may be driven by pain, fear of flare ups, or irreversible visual loss or immunosuppressive treatment [11-13]. In adults with conditions such as uveitis, anxiety and depression is prevalent, even without visual impairment or blindness [14], suggesting that the nature of IODs may be contributing to the prevalence of mental health conditions beyond vision loss alone.

Mental health is not routinely assessed in younger patients with inflammatory eye diseases. In research, where the mental health of children with chronic health conditions is assessed, QoL is typically used instead of a psychiatric assessment due to the complexity of mental health assessments in children [15] and the research is often limited to childhood experiences, but in many cases, the psychological impact of childhood-onset health conditions lasts into adulthood [16,17]. Therefore, it is vital to understand the mental health impact of chronic health conditions throughout childhood, adolescence, and adulthood. However, despite this broader evidence base, there is no reported prevalence of mental health conditions in children, young people, or adults with childhood-onset IODs.

Given the established physiological link between inflammation and depression, and the evidence connecting visual impairment and childhood-onset health conditions to adverse mental health outcomes, we seek to determine whether a similar relationship exists between childhood-onset IODs and mental health conditions. The aim of this systematic review is to determine the prevalence of mental health conditions in children and adults with childhood-onset IODs and to understand the risk factors for mental health conditions and reduced QoL in this population.

The following is the primary systematic review research question: What is the pooled prevalence of mental health disorders in individuals affected by childhood-onset IOD?

The following are the secondary research questions: What is the prevalence of low QoL and well-being in children and adults with childhood-onset IOD? What are the risk factors for adverse mental health and low QoL following a diagnosis of childhood-onset IODs?

## Methods

### Study Design

#### Overview

A systematic review (PROSPERO registration: CRD420251182619) of any studies that report on the prevalence of mental health conditions in people with childhood-onset IOD will be conducted. Studies that evaluate the effectiveness of an intervention will not be included as they are unlikely to report on prevalence. The review will be conducted systematically with a minimum of 2 reviewers at each stage of the process. The full inclusion and exclusion criteria are shown in Textbox 1.

**Inclusion and exclusion criteria.**

**Inclusion criteria**

- Study populations must include individuals with childhood-onset chronic inflammatory ocular diseases (IODs), specifically uveitis, keratitis, scleritis, conjunctivitis, retinitis, optic neuritis, dry eye disease, orbital inflammation, or keratoconjunctivitis.
- Studies must report on the mental health outcomes of children, young people, and adults with childhood onset of these ocular diseases. Studies must specify “childhood onset” or report age of onset <18 years.

**Exclusion criteria**

- Studies that limit study populations to those with multisystem disease within which IODs may manifest.
- Studies that limit reporting of the exposure with resultant inability to identify outcomes among the population with a confirmed diagnosis of IOD.
- Studies that limit study design on the evaluation of the impact of blindness or visual impairment in children.
- Studies that evaluate the impact of an intervention on mental health outcomes of children with IOD.
- Randomized controlled trials in which quality of life is used as an outcome metric.

#### Population

The study will include children, young people, and adults with childhood-onset IOD. A diagnosis of an IOD must have been made for participants before they turned 18 years old. Children and adults who are visually impaired or blind will not be included in this review.

#### Exposure

Eligible ocular diseases include conditions where inflammation is the main pathology including uveitis, keratitis, scleritis, conjunctivitis, retinitis, optic neuritis, dry eye disease, orbital inflammation, or keratoconjunctivitis.

#### Confounders

Factors that may influence the prevalence rates, such as socioeconomic status, ethnicity, medication, frequency of appointments, type of autoimmune condition, and QoL.

#### Outcome

Number or proportion of study population self-reporting (including parent or guardian reporting) or diagnosed with a mental health disorder, low QoL, or impaired well-being as assessed using validated diagnostic or screening tools. Mental health disorders comprise anxiety disorders, depression, bipolar disorder, posttraumatic stress disorder, schizophrenia, eating disorders, attention-deficit/hyperactivity disorder, obsessive compulsive disorder, and disruptive and dissocial disorders.

### Inclusion and Exclusion Criteria

There will be no restrictions on the language, study type, geographic location, or publication date. Studies that are not in English will be translated using “Adobe translate” or Google translate.

### Search Strategy

Searches will be conducted on the PubMed, Cochrane Central Register of Controlled Trials, Embase, Ovid, and PsycArticles databases. Gray literature will also be searched to identify any unpublished papers using Google to search for terms related to the research question and add to record any relevant results. This method was chosen as it has a broad reach.

The first 3 pages of results for each search will be checked for relevant studies. Studies found via gray literature searching will be added to the group of studies to be assessed for eligibility.

The search terms were decided based on the research question and using the Medical Subject Headings (MeSH) thesaurus (Textbox 2).

**Search terms.**

**Search query 1**

- “Uveitis,” “Keratitis,” “Scleritis,” “Conjunctivitis,” “Retinitis,” “Optic Neuritis,” “Dry eye,” “Orbital inflammation,” “Keratoconjunctivitis,” “Ocular diseases”

**Search query 2**

- “Chronic disease,” “Long term care”

**Search query 3**

- “Child,” “Young adult,” “Adolescent,” “Childhood onset”

**Search query 4**

- “Mental health,” “Quality of life,” “Psychological well-being,” “Depression,” “Depressive disorder,” “Anxiety,” “Psychological,” “Emotion”

### Article Selection Process

All search results will be exported to Covidence for review. The articles will be checked initially for duplicates which will be removed. The remaining articles will be screened in a 2-step process. First, 2 authors will screen all the titles and abstracts for eligibility. In the second stage, the same 2 authors will read the full-text versions of the articles to confirm inclusion. Disputes at any stage of the review process will be taken to a third author who will make the final decision.

Articles that are included in the review will then undergo a data extraction process using a form on Microsoft Excel. After piloting with 2 of the authors (SD and AV) and a discussion among the research team, it was decided that the following data will be extracted as shown in Textbox 3.

**Data to be extracted.**

- Study ID, title, author, year of publication, citation, and country
- Study design
- Study type
- Study duration
- Inclusion and exclusion criteria
- Population characteristics—sample size, gender, ethnic diversity, age, concurrent systemic diagnoses, and any other details of study population
- Diagnosis (inflammatory ocular disease)
- Quantitative or mixed methods
- Measurement of mental health conditions (ie, formal or validated diagnostic process, patient-reported outcome measures, imaging, interviews, and self-report?)
- Details of other outcome measures
- Proportions with mental health conditions
- Reported associations between risk factors and outcomes
- Significance

### Risk of Bias and Quality Assessment

The studies that will be included in this review will mainly be observational studies that evaluate the relationship between mental health conditions, QoL, and well-being in people with childhood-onset IODs. Therefore, the risk of bias in nonrandomized studies of exposure tool will be used to examine the strength of evidence included in the study [18]. The tool is designed to help researchers assess the potential effects of environmental, occupational, and behavioral exposures on health in observational studies. Checklists from the Critical Skills Appraisal Programme will be used to assess the quality of the studies included in this review [19]. In the case of no appropriate checklists being available from the Critical Skills Appraisal Programme database, quality assessment checklists from the JBI will be used [20].

### Synthesis Methods

A summary table with the narrative descriptions of the included studies will be reported. A meta-analysis will be conducted with quantitative data from the included studies where possible. All analyses will be performed with R statistical software (version 3.3.2; R Foundation for Statistical Computing), and *P* values <.05 will be used as the threshold for statistical significance. The total prevalence of mental health conditions will be calculated by extracting the number of cases and the total number of participants from each study. The prevalence and odds ratios will be pooled with the random-effects model. This will be more appropriate as the fixed effects model may be too restrictive. Forest plots will be used to summarize individual and pooled prevalence estimates of mental health and QoL. Stratified prevalence will be calculated separately for the risk factors using the same processes. Summary statistics will be expressed as odds ratios and 95% CIs. Heterogeneity between studies will be assessed using the *I^2^* statistic (values of 25%, 50%, and 75% will be considered low, medium, and high heterogeneity, respectively).

### Ethical Considerations

Due to the nature of this systematic review, which involves the analysis of previous studies, rather than inclusion of human or animal participants, approval from a research ethics committee is not required. This systematic review will not use any identifiable information about any of the study participants, and the authors will take necessary precautions to minimize risk.

## Results

Preliminary searches were conducted in October 2025 to establish an approximate number of reports. Formal searches began in November 2025. Data collection and extraction is currently in progress. It is anticipated that the final systematic review will be completed by August 2026.

## Discussion

### Anticipated Findings

This systematic review aims to establish the prevalence of mental health conditions among children and adults diagnosed with IOD. There is a well-established link between inflammation and mental health conditions, with several studies showing that neuroinflammation specifically is associated with increased risk of depression [21-23]. The evidence base of studies focusing on childhood onset of disease appears limited. Even in studies describing the association of mental health conditions and autoimmune diseases, a very limited number report on diseases of childhood onset, and even fewer on the associated ocular diseases. Therefore, this systematic review should provide a much-needed focus on the gaps in the evidence base to identify future research directions. The planned compilation and synthesis may provide findings which indicate potential areas of support for children and families and the identification of risk factors that underpin excessive psychological burden.

These findings may also help identify protective factors that may reduce the likelihood of mental health conditions developing in some children, young people, and adults. Studies involving children, young people, and adults with visual impairments show that ethnicity, social support, and higher socioeconomic status can be protective of mental health conditions and low QoL [10,24-26] The identification of these factors will provide valuable insight for future research into interventions for children, young people, and adults that are diagnosed with IODs.

### Strengths and Limitations

Study strengths comprise, first, novelty and the addressing of an evidence gap: the prevalence of mental health conditions in this population has not been established previously. Studies have reported on the mental health of children and adults with visual impairment, but this study aims to evaluate whether mental health conditions are present in patients with IOD in the absence of established visual loss. Second, the review will include children and adults with childhood-onset IOD. This means that even if mental health conditions were not prevalent in childhood, later presentations will be included.

However, we acknowledge that there are limitations to this review. Not limiting the study to one type of mental health measurement may mean that less sensitive measurement tools have been used. Robust quality assessment of included studies should mitigate against this potential short coming, and heterogeneity in methodology will be analyzed and will be an important finding for the review. Including QoL as a secondary research question and including studies that report on QoL may not accurately measure mental health prevalence. Although QoL measures can capture some subjective experiences, functional burden, and social functioning, it cannot fully substitute a psychiatric assessment. However, studies that use QoL measures will be included as there are limited studies using pediatric mental health measure due to complexities with reporting due to age and stigma [27,28]. The significant gap in the literature supports the need to include both mental health studies and QoL studies to capture the full experiences in this population. Finally, Google was used for the gray literature search. While the use of search engines (eg, Google) is often discouraged due to potentially overwhelming quantities of results, the narrow scope of this topic rendered this approach appropriate, owing to Google’s extensive reach. This will also be mitigated for through the systematic process and the application of the risk of bias assessment for all unpublished studies.

### Conclusions

This systematic review will be the first to outline the prevalence of mental health conditions in children and adults with childhood-onset IOD. Inflammation is a key pathology in depression and anxiety, with a resultant potential risk of poor outcomes among those with IOD. The importance of establishing the prevalence of mental health conditions in this population lies in the impact of these findings on future research and health care policy. The findings of this review will help to establish the risk of a mental health condition and inform the management of children and adults with childhood-onset chronic health conditions.

## Abbreviations

IOD: inflammatory ocular disease
MeSH: Medical Subject Headings
QoL: quality of life

## Data Availability

The datasets generated or analyzed during this study are available from the corresponding author on reasonable request.
